# Food Insecurity and Common Mental Disorders among Ethiopian Youth: Structural Equation Modeling

**DOI:** 10.1371/journal.pone.0165931

**Published:** 2016-11-15

**Authors:** Mulusew G. Jebena, David Lindstrom, Tefera Belachew, Craig Hadley, Carl Lachat, Roos Verstraeten, Nathalie De Cock, Patrick Kolsteren

**Affiliations:** 1 Population and Family Health, Jimma University, Jimma, Ethiopia; 2 Department of Sociology, Brown University, Providence, Rhode Island, United States of America; 3 Deparment of Anthropology, Emory University, Atlanta, Georgia, United States of America; 4 Department of Food Safety and Food Quality,Ghent University, Coupure Links, Ghent, Belgium; 5 Nutrition and Child Health Unit, Department of Public Health, Institute of Tropical Medicine, Antwerp, Belgium; TNO, NETHERLANDS

## Abstract

**Background:**

Although the consequences of food insecurity on physical health and nutritional status of youth living have been reported, its effect on their mental health remains less investigated in developing countries. The aim of this study was to examine the pathways through which food insecurity is associated with poor mental health status among youth living in Ethiopia.

**Methods:**

We used data from Jimma Longitudinal Family Survey of Youth (JLFSY) collected in 2009/10. A total of 1,521 youth were included in the analysis. We measured food insecurity using a 5-items scale and common mental disorders using the 20-item Self-Reporting Questionnaire (SRQ-20). Structural and generalized equation modeling using maximum likelihood estimation method was used to analyze the data.

**Results:**

The prevalence of common mental disorders was 30.8% (95% CI: 28.6, 33.2). Food insecurity was independently associated with common mental disorders (β = 0.323, P<0.05). Most (91.8%) of the effect of food insecurity on common mental disorders was direct and only 8.2% of their relationship was partially mediated by physical health. In addition, poor self-rated health (β = 0.285, P<0.05), high socioeconomic status (β = -0.076, P<0.05), parental education (β = 0.183, P<0.05), living in urban area (β = 0.139, P<0.05), and female-headed household (β = 0.192, P<0.05) were associated with common mental disorders.

**Conclusions:**

Food insecurity is directly associated with common mental disorders among youth in Ethiopia. Interventions that aim to improve mental health status of youth should consider strategies to improve access to sufficient, safe and nutritious food.

## Introduction

Common mental disorders (CMDs) refer to mental problems that may not fall into standard diagnostic criteria. They are characterized by symptoms of sleeplessness, exhaustion, irritability, poor memory, difficulty in concentrating and somatic complaints [1, 2], but may also include disorders classified within the International Classification of Diseases-10 as “neurotic, stress-related, somatoform disorders” and “mood disorders” that usually manifest with the occurrence of a combination of non-specific anxiety, depressive and somatic symptoms [3]. Globally, CMDs account approximately 13% of burden of diseases [4].

Previous studies have shown that the causes of CMDs are multi factorial and include, but are not limited to, biological, social and economic factors [2, 5–10]. Food insecurity is one of the social determinants of mental health reported in both developed and developing countries [7, 11–16]. Food insecurity is defined as lack of access to safe and sufficient food by all people at all times or uncertainty about acquiring acceptable food in socially acceptable ways[17].

The biological and social consequences of food insecurity have been reported among adults, pregnant women, mothers and young children. It compromises intake of energy and nutrients, lowers physical performance, increases risk of chronic conditions and it is also associated with poor nutritional status, developmental outcomes, health and poor cognitive performance [11, 13, 18–20].

**Fig 1:**
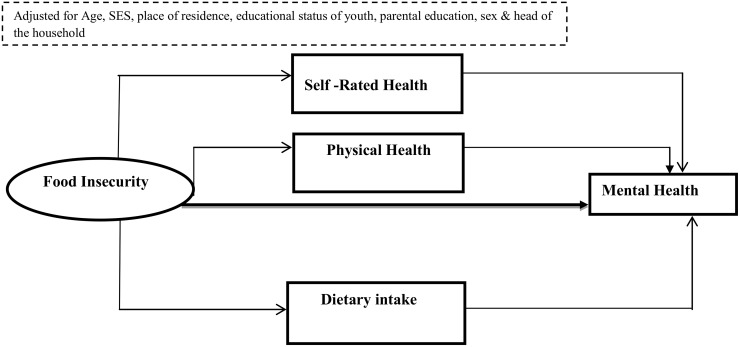
Conceptual framework showing the link between food insecurity and youth common mental disorders, JLFSY, 2015.

A growing body of evidence demonstrates that the consequences of food insecurity extend to mental health problems [21, 22]. Previous studies from developing countries also report the impact of food insecurity on mental health [16, 23–26]. Nonetheless, there are gaps in existing literature. First, available evidence is based on household level food security status and the responses of the head of the household or mothers. Asking a mother or head of the household might be reasonable for young children since intake of food at this age depends on parental factors and their psychopathology (e.g. parental mental health status, feeding practices, caring and parenting style and other familial characteristics)[14, 15]. Effects of food insecurity in later adolescence however, might be independent of family characteristics. In addition, food insecurity measured at the household level may not be linked to an individual own food insecurity experience during adolescence. Therefore, we assumed individual level experience of food insecurity to be a better indicator to predict health effects in individuals rather than relying on the response of the head of the household or only focusing on household level food security status. Secondly, the detrimental effect of food insecurity among youth living in low and middle income countries, where both food insecurity and mental health are common, has been poorly studied. Most of the studies from reported the association of food insecurity with mental health from low and middle income countries have focused on women, children and adults [14, 19, 27, 28]. Thirdly, the mechanisms by which food insecurity is linked in to poor mental health have not well investigated and can be organized in various hypotheses. First, the *biological* perspective assumes that the causality of food insecurity and poor mental health status is mediated by poor nutritional status. For instance, food insecurity affects health either directly or indirectly through poor diet and nutritional status. In turn, diet and nutritional status predict physical health, quality of life and health status [14, 15, 20, 29, 30]. Other studies have also demonstrated that food insecure children have a poorer nutritional intake (e.g. micronutrients, fiber, energy and carbohydrates) [11, 31–33]. Secondly, the *psycho-emotional* perspective assumes that food insecurity leads to psychological problems such as extreme stress, psychological distress that could lead to poor self-rated health status. This poor self-rated health status in turn is associated with poor mental health such as depression. In other words, those who are food insecure could experience negative emotions which are translated inside the body and can contribute to poor mental health [34–37]. As a third hypothesis, the role of poor physical health or exposure to morbidities in mediating the relationship between food insecurity and mental health is proposed. Food insecurity could affect physical health. Poor physical health in turn poor physical health influences mental health. For instance, studies have shown that children in food insecure households are more likely to have frequent stomach-aches, headaches and poor developmental outcomes. This frequent exposure to morbidities in turn impacted their mental health status [12, 20, 22, 30, 38, 39]. Others studies have also argued that food insecurity could be directly linked to poor mental health status independent of other factors and or without involvement of a mediator [20, 40]. For instance, Vozoris NT. et.al. (2003) and Cook et. al. (2004) suggested that food insecurity is associated with adverse health outcomes in children even when it does not involve reductions in the quantity of food intake[19, 30]. This may be due to both food insecurity and children’s poor mental health have common causes such as parental psychopathology, low socio economic status [15, 16, 38, 40, 41] (Fig 1).

Although associations of food insecurity and mental health have been reported by many literature, few of them have tested all the model components simultaneously. As both food insecurity and mental health are latent constructs, model that take in to account the latent structure should be used. Therefore, the aim of this study was to examine the pathways through which food insecurity is linked to youth mental health after controlling for other factors. It is expected that findings of this study will improve planning of interventions for prevention of both mental health and food insecurity status.

## Materials and Methods

### Study setting, sampling and design

We used third round data from the Jimma Longitudinal Family Survey of Youth (JLFSY), which is a representative cohort of adolescents living in Jimma Zone, Southwest Ethiopia. The JLFSY study began in 2005 and is designed to examine the social and economic determinants of adolescent’s health and well-being. The study area encompassed rural, semi-urban, and urban communities to represent a range of agro ecological zones and development contexts. A total of six kebeles’ of Jimma Town, including three kebeles of nearby semi urban towns, and 18 rural kebeles around each town, were purposively selected. A kebele is the smallest administrative unit of Ethiopia similar to a ward, a neighborhood or a localized and delimited group of people. Stratified random sampling was used to select household for inclusion in each site.

A two-stage sampling plan was used to select eligible adolescents for the study. At the first stage, households were randomly sampled from each study site. The sample size in each site was determined by the relative proportion of the study population in the site and the overall target sample size. At this stage a total of 3700 households with at least one male or one female adolescent were identified. In the second stage, one adolescent aged 13 to 17 years was randomly selected from each household using a Kish Table [13, 42]. Data were collected every one and half years, and the data collection process took 2–3 months of duration. A total of 2,084 youths (1,059 boys and 1,025 girls) were followed over the last 10 years. The present analysis was cross sectional and used only the third round data collected in the year 2009/10. A total of 1,521 youths aged 17–21 years with no missing information on the variable of interest were included in the analyses. Ethical approval was obtained from Jimma University and Brown University (USA). Informed verbal informed consent was obtained from both head of the households and adolescents before the interview or measurement as approved by the ethics committees.

### Data collection methods

Data were collected using structured questionnaires developed in English and then translated into Amharic and Afan Oromo languages. The Amharic and Afan Oromo versions were back-translated into English to verify accuracy. Twelve interviewers and two supervisors who completed secondary level education were recruited considering their prior experiences and fluency in Amharic and Afan Oromo languages.

The head of the household responded to the household questionnaire. The questionnaire for adolescents focused on issues related to personal experiences of food insecurity, education, health, nutrition and anthropometric measurements. The interview was conducted in a private place by an interviewer of the same sex as the respondent. Adolescents were interviewed after the interview with the household head was completed. The interviewers remained with the same households and adolescent respondents throughout the study period. Prior to pre-testing the forms, the interviewers and supervisors received one week of intensive training, and questionnaires were pre-tested in the study area to identify problems related to content, wording and formatting. Supervisors kept track of the field procedures and checked the completed questionnaires every day to ensure accuracy of the data collected.

### Estimates of scale reliability

Both exploratory and confirmatory factor analyses were computed to identify factor variances explaining the most and to check item loadings, scale reliability and model fitness respectively. To estimate scale reliability, Cronbach's alpha was computed after confirmatory factor analysis was performed using the following formula:

$$\rho =\frac{\sum {\left(\lambda i\right)}^{2}}{\sum {\left(\lambda i\right)}^{2}+\sum \theta ii+2\sum \theta ij}$$

where Σ(*λi*)^2^ is the squared sum of the unstandardized loadings called lambdas, Σ*θii* is the sum of unstandardized error variances called thetas, the 2 Σ*θij* is two times the sum of unstandardized covariance of the errors, if there is correlated error in the model. When there are no correlated errors the equation reduces to $\rho =\frac{\sum {\left(\lambda i\right)}^{2}}{\sum {\left(\lambda i\right)}^{2}+\sum \theta ii}$

### Measurements

#### Food insecurity

Food insecurity was measured using a questionnaire previously developed, adapted and tested in the same study settings [13, 43–48]. Respondents were told to think about their own food insecurity experience and then were asked whether they had experienced different conditions in the last three months (S1 Table). Food insecurity was treated as latent exogenous exposure variable in the structural equation modeling.

#### Physical health

Physical health status was measured using a 10 item questionnaire asking whether they had experienced health morbidities in the past two weeks prior to the data collection period (S1 Table). Responses to these morbidity questions were binary recoded “1” for Yes and “0” for No. A row sum score was computed and log transformed. Thus, we used the log transformed physical health score and treated it as a continuous mediator variable.

#### Self-rated health

Youth's self-rated health status was assessed using a one-item question where individuals were asked *In general*, *how would you rate your health today*? Possible responses range from very good, good, moderate, to poor. Since responses were skewed, we used a natural logarithm transformation and treated it as continuous variable when it was included in the model (S1 Table).

### Mental health

To assess mental health status (common mental disorders), we used Self-Reporting Questionnaire (SRQ-20) (S1 Table). The SRQ is a 20-item retrospective survey with yes/no answers that measures psychiatric symptomatology. Development of the SRQ was reported by the World Health Organization[49, 50]. The SRQ includes both somatic items (e.g., headaches, loss of appetite, tiredness) and psychological items (e.g., feeling unhappy, nervous, and worthless). It is used throughout low-and middle-income countries, including Ethiopia and many other African settings [10, 16, 49, 51–53].

**Fig 2:**
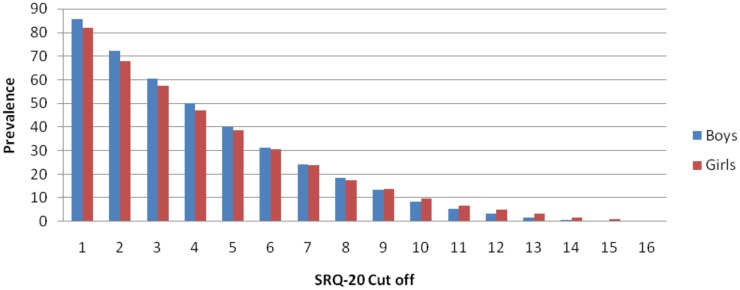
Prevalence of SRQ-20 Using Different Cut-Points by Gender, JLFSY 2015.

Fig 2 summarizes the prevalence of common mental disorders by sex using different cut points. To determine the cases of poor mental health, a cut- off value of ≥6 was used in Ethiopia [10] although a number of studies in sub-Saharan Africa suggested a range of cut-off points[51, 52, 54]. However, the SRQ-20 tool was not validated for the young population in Ethiopia. Thus, we computed raw SRQ-score assuming each item had equal weights. We also generated a "mental health index” using principal component analysis, assuming that each item is weighed according to its contribution to the mental health status. Spearman’s correlation between the mental health index and raw score was high (rho > 0.99, P< 0.001). We therefore chose to use mental health index and treated it as a continuous variable in the structural equation modeling (SEM) analysis.

### Diet diversity

Dietary diversity was assessed using a food-frequency questionnaire containing 30-food items [55–60]. Participants were asked to report the frequency of typical consumption of each food using the past 3 months as a reference. Participants were coded as a “consumer” of a food item if they had consumed the food item at least once per week. Factor analysis was performed to generate a dietary diversity score where the higher score corresponds to higher level of diet diversity. The detailed procedures were described in previously published work from similar data sets [18, 44, 60].

### Covariates

Potential covariates were identified *a priori*. These included age, gender, female-headed household, parental education, educational status of youth, household wealth and place of residence. We computed the index of household socioeconomic status based on household ownership of 21 items. The index included items such as a functioning radio, television, cooking stove, various furniture items, and farming implements [13]. The index ranges from 0 (not owning any of the items) to 21 (owning all of the items). In addition, principal component analysis was used to construct the wealth index, which was treated as continuous variable. Internal consistency of the items was adequate (Cronbach’s alpha = 0.83).

### Data analysis

The data entered were verified for missing values, outliers, and normality prior to analysis using Stata Version 13.0 (Stata, Corp). Descriptive data were reported as percentage, means and Standard deviations (SD).

For each of the exogenous and endogenous mediators and endogenous outcome variables, reliability of each item was tested in respective of its constructs. Factor and principle components analysis were used. Overall scale modification and removal of items was made based on factor scoring, error terms and factor loading criteria. For each construct, only items having greater than 0.4 factors loading were included and those items that were cross-loaded on more than two factors were omitted. Finally, confirmatory factor analysis was done for the measurement model to evaluate the relationship between each construct.

Both SEM and generalized structural equation modeling (GSEM) using maximum likelihood estimation method were employed based on a conceptual hierarchical modeling framework after adjusting for covariates[14, 61]. First, after controlling for covariates, the independent effect of food insecurity on mental health was assessed (Model I). We added self-rated health status to evaluate its effect on mental health (Model II). In Model III, the effect of dietary diversity was estimated. Path coefficients for all parameters and their significance levels were determined at each step. Finally, the mediation effect of physical health was tested (Model IV). The goodness of fit test was checked using χ²-test statistic, Comparative Fit Index (CFI), Root Mean Square Error of Approximation (RMSEA), Standardized Root Mean square Residual (SRMR), Non Normed Fitted Index (NNFI), Pclose and coefficient of determination (R^2^). Accordingly, CFI/NNFI value of >0.90, RMSEA< = 0.08, Pclose>0.05, and SRMR<0.08 were used as standard criterion to determine model fitness. We improved the model using modification of indices and removing non-significant paths and comparing the fit of multiple models using the Akaike Information Criteria [62].

To test the effect of mediators, we followed procedures by Baron and Kenny (1986). First, we observed the significant association between food insecurity and mental health. Then we assessed whether food insecurity predicted mediating variables (physical health, self-rated health and diet diversity score); and finally we checked independent association of mediators on mental health status [63, 64].

The SEM for the mediation model for the i^th^ subject was given by:

$$Z_{i}=\beta_{0}+\beta_{xz}X_{i}+\varepsilon_{zi}\;and\;Y_{i}=\gamma_{0}+\gamma_{zy}Z_{i}+\gamma_{xy}x_{i}+\varepsilon_{yi}$$

Where **Z**_**i**_ was the physical health, self-rated health and diet diversity score, x_i_ was food insecurity status, and **Y**_**i**_ was our outcome of interest (CMDs).

**Fig 3:**
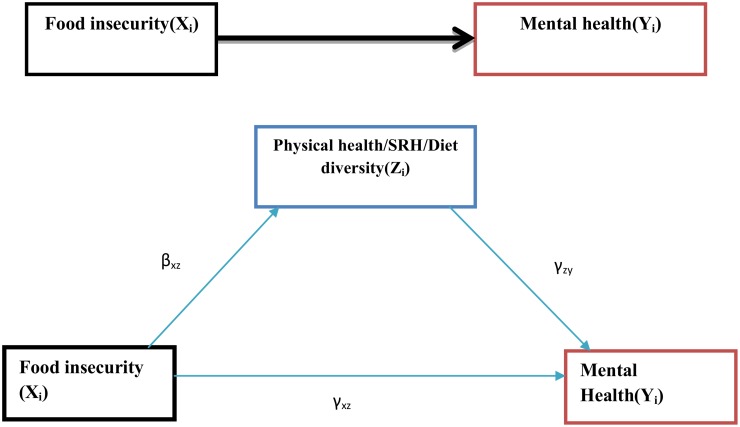
Shows the effect of food insecurity on youth common mental health status, the mediation role of physical health, self-rated health and diet diversity, JLFYS, Southwest Ethiopia, 2015.

We assumed that the error terms (**ε**_**zi**_, **ε**_**yi**_) were uncorrelated and normally distributed. The direct effect was the pathway from the food insecurity to mental health while controlling for the physical health, self-rated health and diet diversity score (i.e. **γ**_**xy**_ is the direct effect). Similarly, the indirect effect described the pathway from the food insecurity to the mental health through physical health, self-rated health and diet diversity. This path was represented by the product of **β**_**xz**_ and **γ**_**zy**_. Finally, the total effect was the sum of the direct and indirect effects of the exogenous variable on the outcome, **γ**_**xy**_ + **β**_xz_*γ_**zy**_ [65]. Using the criteria set by Baron and Kenny [63, 64], interpretation of the role of mediators was explained using standardized estimates of both direct and indirect paths. While estimating the indirect effect, the full mediation was achieved if both were significant and if the direct effect was not significant and close to zero. If that was not fulfilled, partial mediation was reported (Fig 3).

## Results

### Socio-demographic characteristics

A total of 1,521 of sample were included in the main analysis. Out of these, 57.2% were males and the mean (±SD) age of the respondents was 18.75 years (±1.37) for males and 18.73 years (±1.35) for females. The CMDs was 30.85% (95% CI: 28.58, 33.22). There was a statistical significance difference in CMDs by socio economic status (P = 0.014), education (P = 0.052), parental education (P<0.0001), place of residence (P<0.0001) and head of the household (P<0.0001) (Table 1).

**Table 1:** Socio demographic characteristics of Youth (n = 1,521), Jimma Longitudinal Family Youth Survey (JLFSY), Southwest Ethiopia, 2015.

Characteristics	CMDs		P
	Yes	No	
Sex			0.748
Male	271(31.19)	598(68.81)	
Female	198(30.41)	453(69.59)	
Religion			0.002
Muslim	250(18.15)	659(72.50)	
Orthodox	189(36.35)	331(63.65)	
Others*	30(32.97)	61 (67.03)	
Age Mean (±SD)	18.74(±1.307)	18.75(±1.380)	
Educational status			
No schooling	29(23.02)	97(76.98)	0.057
Primary	84(28.38)	212(71.62)	
Secondary and above	356(32.42)	742(67.58)	
Parental education			
No schooling	156(23.85)	498(76.15)	<0.001
Primary	120(37.74)	198(62.26)	
Secondary and above	193(35.22)	355(64.14)	
Residence			
Urban	211(39.22)	327(60.78)	<0.001
Semi-Urban	142(32.49)	295(67.51)	
Rural	116(21.28)	429(78.72)	
SES			<0.001
Low SES	177 (39.86)	267 (60.14)	
Average SES	165 (31.61)	357 (68.39)	
High SES	127(22.92)	427 (77.08)	
Head of the household			<0.001
Female	117(43.98)	149(56.02)	
Male	352(28.07)	902(71.93)	

* *Others include Catholics*, *Protestant*; *CMD* = *Common Mental Disorders*, *SES* = *Socio Economic Status*

### Internal consistency, model fitness and factor loadings

In the exploratory factor analysis of food insecurity, the first factor explained 80.9% of the variation in answers to these questions. Factor loading of the items varied from 0.57 to 0.93 and its coefficient of reliability was also good (Cronbach's alpha = 0.85). Similarly, the exploratory factor analysis for physical health measures showed that 70.4% of the proportion of variance was explained by the first factor and the reliability test of items was acceptable (Cronbach's alpha = 0.77). Exploratory factor analysis of mental health items also showed that the first factor explained 76.9% of the total variance. After confirmatory factor analysis, the item reliability scale was also acceptable (Cronbach's alpha = 0.78) (Table 2).

**Table 2:** Internal consistencies, model fitness and factor loadings of food insecurity, physical health and mental health items, JLFSY, Southwest Ethiopia, 2015.

Food insecurity	Factor loadings	Goodness of fit
In the last three months, how many days did you worry that you would run out of food or not have enough money to buy food?	0.9059	RMSEA = 0.07; LR,X^2^pvalue = 0.003 PClose = 0.147 CFI = 0.997 NNFI = 0.984 CD = 94.9 SRMR = 0.005 Cronbach’s alpha = 0.85
In the last three months, how many days have you had to reduce the number of meals eaten in a day, because of shortages of food or money?	0.9196	
In the last three months, how many days have you had to reduce the size of meals eaten in a day, because of shortages of food or money?	0.9373	
In the last three months, how many days have you had to spend the whole day without eating, because of shortages of food or money?	0.5746	
In the last three months, how many days have you had to ask for food or money to buy food?	0.6133	
**Physical health**		
Think about the last time you were sick, did you have any of the following symptoms? (possible response is 1. yes 0.No)		RMSEA = 0.08 PClose = 0.000 LR,X^2^ pvalue = 0.000 NNFI = 0.892 CFI = 0.931 SRMR = 0.039 CD = 80.3 Cronbach’s alpha = 0.77
1. Fever (hot body)	0.7596	
2. Cough	0.5973	
3. Difficult or fast breathing	0.5939	
4. Diarrheal	0.5025	
5. Vomiting	0.6028	
6. Unable to eat or drink	0.7371	
7. Abdominal pain	0.6996	
8. Extreme sadness/worry	0.5744	
**Mental Health**		
Now I would like to ask you some more questions about your health and how you have felt during the last 30 days. For these questions we will use the red and green or yes and no section of the response card. (Responses: 1. Yes 0. No)		
SRQ1. In the last 30 days do you often have headaches?	0.4054	RMSEA = 0.045 LR,X^2^ pvalue = 0.000 PClose = 0.97 CFI = 0.887 NNFI = 0.870 SRMR = 0.037 CD = 77.8 Cronbach’s alpha = 0.78
SRQ2. In the last 30 days is your appetite poor?	0.4284	
SRQ3. In the last 30 days do you have problems sleeping?	0.4893	
SRQ4. In the last 30 days are you easily frightened?	0.4742	
SRQ5. In the last 30 days do your hands shake?	0.5068	
SRQ6. In the last 30 days do you feel nervous, tense or worried?	0.4018	
SRQ8. In the last 30 days do you have trouble thinking clearly?	0.5558	
SRQ9. In the last 30 days do you feel unhappy?	0.5351	
SRQ10. In the last 30 days do you cry easily?	0.5225	
SRQ11. In the last 30 days do you find it difficult to enjoy your daily activities?	0.5065	
SRQ12. In the last 30 days do you find it difficult to make decisions?	0.5115	
SRQ13. In the last 30 days are you not completing well your work?	0.4399	
SRQ14. In the last 30 days are you unable to play a useful role in other people’s lives?	0.4485	
SRQ15. In the last 30 days have you lost interest in things?	0.4314	
SRQ17. In the last 30 days have you thought of ending your life?	0.5138	
SRQ19. In the last 30 days do you have uncomfortable feelings in your stomach?	0.4457	

**Note: JLFSY = Jimma Longitudinal Family Survey of Youth**; LR X^2^ = Likelihood Ratio Chi square test; CFI = Comparative Fit Index; RMSEA = Root Mean Square Error of Approximation; SRMR = Standardized Root Mean square Residual; NNFI = Non Normed Fitted Index; CD = Coefficient of Determination; SRQ = The Self Reporting Questionnaire

### Confirmatory factor analysis

**Fig 4:**
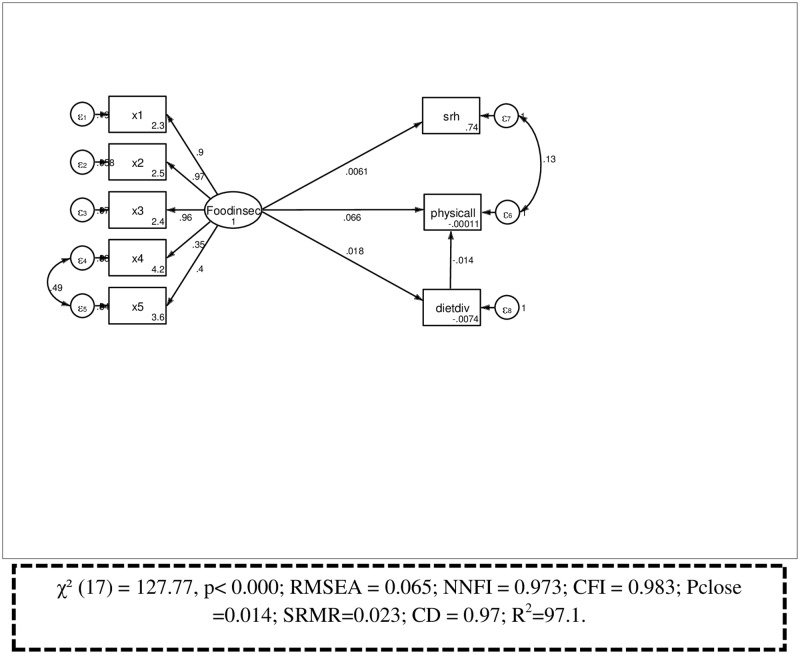
Measurement model of constructs, Jimma Longitudinal Family Survey of Youth, Southwest Ethiopia, 2015.

The confirmatory factor loadings for the whole measurement models were significant at the 0.05 level. The overall fit of the measurement model was adequate (χ² (18) = 128.686, p< 0.0001; RMSEA = 0.064; NNFI = 0.973; CFI = 0.983; Pclose = 0.014; SRMR = 0.023; CD = 0.97; R^2^ = 97.1; (Fig 4). Multicollinearity tests were also acceptable (mean Variance inflation factor = 1.29, minimum tolerance = 0.478; condition number = 15.95).

### Structural equation model

**Fig 5:**
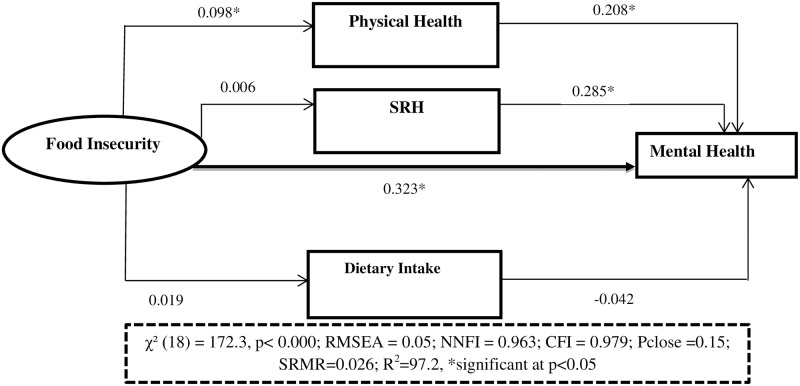
Full structural equation model showing the association between food insecurity and mental health, JLFYS, 2015.

The results of the SEM are depicted in Table 3 using estimates of path coefficients and standard errors with their p values. The overall fit of the structural model predicting mental health was adequate based on the standard fit criteria (χ² (31) = 172.33, P< 0.001; RMSEA = 0.05; NNFI = 0.963; CFI = 0.979; Pclose = 0.15 CD = 0.972). Respondents who experienced food insecurity had a higher likelihood of having CMDs (β = 0.323, P = <0.05) (Fig 5).

**Table 3:** Unstandardized Path coefficient estimates of food insecurity on youth common mental disorders, JLFSY, Southwest Ethiopia,2015.

Structural and measurement model	Model I Path coefficient (SE)		Model II Path coefficient (SE)		Model III Path coefficient (SE)		Model IV Path coefficient (SE)	
	SEM	GSEM	SEM	GSEM	SEM	GSEM	SEM	GSEM
**Step I. Foodinsec—>mental health (Adjusted for covariates)**	0.355(.050)*	0.344 (.050)*	0.386 (.050)*	0.342 (.050)*	0.355(.050)*	0.343 (.050)*	0.330 (.049)*	0.323 (.049)*
Age	0.010 (.018)	0.006 (.018)	0.009 (.018)	0.005(.018)	0.009 (.018)	0.005(.018)	0.013 (.017)	0.009(.018)
SES(low)	-0.129 (.025)*	-0.089(.026)*	-0.130 (.024)*	-0.090 (.025)*	-0.128(.025)*	-0.088(.025)*	-0.110(.024)*	-0.076(.025)*
Sex(female)		-0.031 (.050)		-0.032(.050)		-0.038(.050)		-0.028(.049)
Female Headed household		0.190(.067)*		0.194 (.067)*		0.197(.067)*		0.192 (.065)*
Parental education(Primary)		0.179 (.069)*		0.184 (.060)*		0.189(.060)*		0.183 (.059)*
Education(primary)		-0.011 (.064)		-0.009 (.063)		-0.006 (.063)		-0.033 (.062)
Residence (urban)		0.190(.054)*		0.192 (.054)*		.193(.054)*		0.139(.053)*
**Step II. Model III + SRH**			0.362 (.066)*	0.361 (.065)*	0.360(.066)*	0.360 (.065)*	0.292 (.065)*	0.285(.064)*
Foodinsec—>SRH			0.005(.019)	0.004 (.022)	0.005 (.019)	0.006 (.022)	0.005 (.019)	0.006 (.019)
**Step IV. Model III+ DDS**					-0.038 (.024)	-0.045 (.024)	-0.036 (.023)	-0.042 (.027)
Foodinsec—>DDS					0.044 (.052)	0.019 (.052)	0.044 (.052)	0.019 (.052)
**Step V. Model IV+ Physical health**							0.218 (.024)*	0.208 (.028)*
Foodinsec—>physical health							0.113 (.051)*	0.098 (.051)*
**Goodness of fit**								
Likelihood ratio(x^2^)(df)	115.01(16)		138.10(20)		142.67(25)		172.33(31)	
RMSEA	0.06		0.06		0.05		0.05	
PClose	0.02		0.02		0.14		0.15	
CFI	.985		0.982		0.982		0.979	
NNFI	0.974		0.969		0.968		0.963	
SRMR	0.018		0.02		0.021		0.026	
CD(R^2^)	0.972		0.972		0.972		0.972	

*Note*: *SEM = Structural Equation Model with Maximum Likelihood estimation Method; GSEM = Generalized Structural Equation Model using Maximum Likelihood Estimation Method; SE = Standard Error*; JLFSY = Jimma Longitudinal Family Survey of Youth; LR X^2^ = Likelihood Ratio Chi square test; CFI = Comparative Fit Index; RMSEA = Root Mean Square Error of Approximation; SRMR = Standardized Root Mean square Residual; NNFI = Non Normed Fitted Index; CD = Coefficient of Determination; SRH = Self Rated Health; DDS = Dietary Diversity Score; Foodinsec = Food insecurity; df = Degree of freedom

* *Significant at P <0*.*05*

In this analysis, the pathway between food insecurity and physical health status was statistically significant (β = 0.11, P<0.05) and the pathway between physical health and mental health status of youth was also significant (β = 0.21, P<0.05). However, there was no significant association between food insecurity and dietary intake (β = 0.02, P>0.05) or between dietary intake and mental health ((β = -0.04, P>0.05). Similarly, the effect of food insecurity on self-rated health status was not observed (β = 0.01, P>0.05), although the pathway between self-rated health and mental health status was significant (β = 0.28, P<0.05). In addition, this study also showed socioeconomic status (β-0.08, P<0.05), parental educational (β = 0.18, P<0.05), living in urban areas (β = 0.14, P<0.05), and living in female headed household (β = 0.19, P<0.05) were associated with CMDs. As the wealth of household increased, the likelihood of common mental disorders decreased. Youths living in less educated family had a greater likelihood of having common mental disorders. Youths living in urban areas had higher chance of developing common mental disorders than their rural counterparts (Table 3).

The total effect of food insecurity on mental health was 0.192. The direct component of this total effect was 0.176, or in other words, 0.176 /0.192 = 0.917 or 91.8% of the effect of food insecurity on mental health is direct. By contrast, the indirect effect was 0.016, so that 8.2% of the effect of food insecurity was partially mediated by physical health (Table 4).

**Table 4:** Estimation of direct, indirect and total effect of food insecurity on youth common mental disorders, JLFSY, Southwest Ethiopia 2015.

Mental health status	Direct effect (standardized β coefficient			Indirect effect (standaridzed β coefficient			Total effect (standaridzed β-coefficient		
		Z	P>|Z|		Z	P>|Z|		Z	P>|Z|
Physical health	0.24	9.86	<0.001				0.24	9.86	<0.001
Food insecurity	0.18	7.07	<0.001	.0158425	2.45	0.014	0.19	7.48	<0.001
Foodinsec→physical Health	0.07	2.53	0.012				0.07	2.53	0.010

## Discussion

There is a growing body of evidence demonstrating an association between food insecurity and mental health among children and adults [14, 16, 19, 27, 28]. Although there are few studies, we expected that the underlying relationships between food insecurity and mental health would also hold true for youth. This paper adds values to prior knowledge by exploring the relationship between food insecurity and mental health during late adolescence and early adulthood in a context of low and middle income countries.

In the present study, the prevalence of common mental disorders was 30.8%. This was much higher than in previous studies conducted in Ethiopia, which reported 21.6% among university students [66], 25.8% among adult communities in Jimma Zone [67], and 17.7% among adults living in Addis Ababa [5]. The observed prevalence however, was lower when compared to the studies done among university students in Gonder and Hawassa which reported 40.6% and 49.1% prevalence of common mental disorders respectively [68, 69]. The differences might be due to the cut-off points used to compute prevalence estimates of CMDs. For instance, studies in sub-Saharan Africa have used a range of cut-off points (commonly they use 8, 4, 11 and 7) [51, 52, 54, 67, 68, 70].

Food insecurity was associated with CMDs independent of other covariates. The hypothesized mechanisms by which food insecurity is linked to mental health range from biological pathways, psycho emotional dimensions, parenting skills, and exposure to physical illness [11, 19, 71]. In the present study the pathway between food insecurity and mental health was mainly direct. In addition, this study revealed that physical health has also mediated the relationship between food insecurity and mental health, though this association explained a very small percentage (8.2%). Similar studies have reported the mediating role of physical illness [12, 20, 22, 26, 39, 40, 72]. In contrast, some studies reported other explanatory variables, such as parental mental health status, parenting style, and dietary quality as mediators of the relationship [73–76]. These variables were not collected in our study.

In the present study, there was no observed effect of food insecurity on mental health through reduced dietary intake. However, there are studies that reported food insecurity status would result in lower intake of healthy foods and nutrients, which would ultimately result in changes in health status [77–81]. This discrepancy might be due to differences in grouping, selection of foods, cutoff points, classification systems, measurement tools and approaches between the studies. For instance, some use simple counts of foods or food groups while others take in to consideration the number of servings of different food groups in grouping foods. There are also different types of measurement approaches (e.g. some measures overall variety score (simple count of food items) and others focused on variety score among major food groups). The disparities in the time of reference period for measurement might also affect the results. Future research is required to understand the meditation role of low dietary intake in explaining the causal pathway between adolescent food insecurity and mental health.

Other social determinants of health such as low socio economic status [20], are also significantly associated with CMDs. Previous studies showed that mental disorders are inversely related to income and occur more commonly in families experiencing poverty [12, 14, 20, 26, 29]. The present study also revealed youths living in families with higher income were less likely to have mental disorders compared with those living in lower income households. This might support the social causation theory that tried to explain a wide array of mechanisms through which socio economic status might affect psychopathology. The social causation theory argues that the conditions of life are associated with low socio economic markedly increase the risk of mental disorders either directly and or indirectly through social exclusion, heightened stress, decrease social capital, food insecurity and increased risk of violence [82, 83]. This might highlight the importance of early intervention on social determinants of health such as food insecurity before it persists into adulthood.

The present study also reported a higher proportion of common mental disorders among youth living in urban areas compared to their rural counterparts. A similar finding was observed from studies done in Goa, India [84], Addis Ababa [5] and Malawi [85], showing that those living in urban areas had a higher prevalence of CMDs compared to those living in rural areas. This might be due to rapid changes in social structures, including a highly pressurized living style due to economic vulnerability, job satisfaction, social circumstances and type of employment that could influence psychological conditions. Critical investigation of the effect of urbanization on mental health status in the time of social and economic transformation is needed to explore this association further.

This study has some strengths. To the best of the authors’ knowledge, this is the first report attempting to employ the use of structural equation model to understand the link between food insecurity and CMDs among youth living in Ethiopia. The study also relatively used large sample size. The findings contribute to growing scientific evidence on the impact of food insecurity on the well-being of youth by estimating direct, indirect and the total effect of food insecurity on their mental health status. Although the model does not make causal assertion, it assesses the relationships with mental health conditions. In addition, most of the previous studies that uncovered the effect of food insecurity on youth mental health were from developed countries, and the issues in low- and middle-income countries have been underrepresented. However, this study has some limitations. The first one is the inherent limitation of a cross sectional study design in drawing causal inferences and temporality. We cannot rule out reverse causality, as CMDs are known to produce food insecurity vice versa. Secondly, although we tried to control our analyses for covariates, we cannot entirely rule out the possibility of other confounders and or other explanatory models in determining the association between food insecurity and mental health status. We only assessed a limited set of variables that could explain their relationships. The hypothesized association is not the only model that could be used to examine the link between food insecurity and mental health in the youth population. Alternative models (e.g. adding parental psychopathology) could be used to explore other relationships.

We have both unexplained variance in measuring both food insecurity (e_1_) and mental health measures (e_2_). We hope that there are various variables in common that will influence both food insecurity and mental health, e.g. economic hardship, parental mental health problems, depression status of the mothers, poverty, and parenting style. One way to fit the model is to use instrumental variables that directly influence food insecurity but do not directly influence mental health and vice versa (then to use the predicted values to estimate the effect of food insecurity on mental health or the effect of mental health on food insecurity). These variables were not present in the data set. Readers should take the issues of model specification and endogeneity (omitted variable bias) into account.

## Conclusions

In this study, food insecurity was associated with common mental disorders among youth in Ethiopia. The pathway was mainly direct, suggesting that lack of access to reliable and sufficient amounts of food not only affected youths’ physical health and nutritional status but also their mental health. The implications of these findings are far-reaching. First, exploring such evidence might indicates the social inequalities at earliest age and or the long-term trajectories of food insecurity that could persist into poor health status in adulthood. Second, this evidence could direct program planners, policy makers to design proper interventions for prevention of both mental health and food insecurity status. It gives insights on the importance of considering strategies to improve access to sufficient, safe, and nutritious food to curtail the detrimental effect of food insecurity on physical and mental health of youth. Further analysis will aim to verify these results with additional sources of data to see the longitudinal effect of exposures to food insecurity on mental health, and examine other plausible models to better understand the mechanism by which food insecurity is linked to poor mental health. Additional investigation is also recommended to verify the mediation role of time varying measures, whether changes in the mediator are more likely to precede the change in mental health status, and to what extent temporal order of change over time leads to plausible conclusions about the mediation and their relationship.

## Supporting Information

S1 Table Lists of questions to measure food insecurity, physical health, self-rated health and mental health status, JLFYS, Southwestern Ethiopia, 2015. (DOCX)
