# Supplementary material for: Food Insecurity and Common Mental Disorders among Ethiopian Youth: Structural Equation Modeling
Source: PLoS One. 2016 Nov 15;11(11):e0165931. doi: 10.1371/journal.pone.0165931 (PMC5113011; doi:10.1371/journal.pone.0165931)
Supplement: S1 Table — (DOCX) [file pone.0165931.s001.docx]

**S1 Table. Lists of questions to measure Food insecurity, physical health, self rated health and mental health status, JLFYS, Southwestern Ethiopia, 2015**

| **Variables** | **Lists of Questions** |
| --- | --- |
| Food insecurity | 1. In the last three months, how many days did you worry that you would run out of food or not have enough money to buy food?   **Responses:**   1. Never 2. 1-7 days 3. 8-21 days 4. More than 21 days |
|  | 1. In the last three months, how many days have you had to reduce the number of meals eaten in a day, because of shortages of food or money?   **Responses:**   1. Never 2. 1-7 days 3. 8-21 days 4. More than 21 days |
|  | 1. In the last three months, how many days have you had to reduce the size of meals eaten in a day, because of shortages of food or money?   **Responses:**  1. Never 2. 1-7 days 3. 8-21 days 4. More than 21 days |
|  | 1. In the last three months, how many days have you had to spend the whole day without eating, because of shortages of food or money?   **Responses:**  1. Never 2. 1-7 days 3. 8-21 days 4. More than 21 days |
|  | 1. In the last three months, how many days have you had to ask for food or money to buy food?   **Responses:**  1. Never 2. 1-7 days 3. 8-21 days 4. More than 21 days |
| Self Rated Health | In general, how would you rate your health today?  **Responses**: 1. Very good 2. Good 3. Fair 4. Bad |
| Physical Health | Think about the last time you were sick, did you have any of the following symptoms? |
|  | 1. Fever (hot body) Responses: 1.Yes 0.No |
|  | 2. Cough Responses 1.Yes 0.No |
|  | 3. Difficult or fast breathing Responses 1.Yes 0.No |
|  | 4. Diarrheal Responses 1.Yes 0.No |
|  | 5. Vomiting responses 1.Yes 0.No |
|  | 6. Unable to eat or drink 1.Yes 0.No |
|  | 7. Abdominal pain responses 1.Yes 0.No |
|  | 8. Genital discharge or ulcer responses 1.Yes 0.No |
|  | 9. Depression/extreme sadness/worry responses 1.Yes 0.No |
|  | 10. In the past six months have you had an injury? responses 1.Yes 0.No |
| Mental health | Now I would like to ask you some more questions about your health and how you have felt during the last 30 days. For these questions we will use the red and green or yes and no section of the response card. (Responses: 1. Yes 0. No) |
|  | 1. In the last 30 days do you often have headaches? |
|  | 2. In the last 30 days is your appetite poor? |
|  | 3. In the last 30 days do you have problems sleeping? |
|  | 4. In the last 30 days are you easily frightened? |
|  | 5. In the last 30 days do your hands shake? |
|  | 6. In the last 30 days do you feel nervous, tense or worried? |
|  | 7. In the last 30 days is your digestion poor? |
|  | 8. In the last 30 days do you have trouble thinking clearly? |
|  | 9. In the last 30 days do you feel unhappy? |
|  | 10. In the last 30 days do you cry easily? |
|  | 11. In the last 30 days do you find it difficult to enjoy your daily activities? |
|  | 12. In the last 30 days do you find it difficult to make decisions? |
|  | 13. In the last 30 days are you not completing well your work? |
|  | 14. In the last 30 days are you unable to play a useful role in other people’s lives? |
|  | 15. In the last 30 days have you lost interest in things? |
|  | 16. In the last 30 days do you feel that you are a worthless person? |
|  | 17. In the last 30 days have you thought of ending your life? |
|  | 18. In the last 30 days do you feel tired all the time? |
|  | 19. In the last 30 days do you have uncomfortable feelings in your stomach? |
|  | 20. In the last 30 days are you easily tired? |
